# Malarial Fever as Suppressed, with Illustrative Cases

**Published:** 1884-07

**Authors:** John Hall

**Affiliations:** Toronto, Canada


### MALARIAL FEVER AS SUPPRESSED, WITH ILLUSTRATIVE CASES.

JOHN HALL, M. D., TORONTO, CANADA.

[*Read before I. H. A., June 13th, 1884.*]

On malaria and its manifestations much has been said and written, not unfrequently with evidences of skill and research. Its origin and nature have shared largely in the investigation; its relation to temperaments and sex, and influence on other diseases, such as phthisis, pneumonia, and affections of the nervous system, all these have been more or less ably discussed, not omitting that formidable condition known as malarial cachexia, while little has been elicited on the possibility of suppressing by improper medication the primary symptoms of malarial poisoning, and of thereby engendering other diseases; this alarming possibility, with allusions to some grave consequences and hints at remedial measures, will form the subject of this paper, in the elucidation of which I shall assume as accepted by all Hahnemannians the following propositions:

1. That the outward, visible, or sensational symptoms of a malady are not the disease proper.

2. Such external or visible manifestations are the result of an effort of nature to *eject* the disease proper from the organism.

3. Any attempt to arrest or check such symptoms by other means than a remedy capable of inducing the nearest resemblance to the whole will not cure the internal disease.

4. To check the outward expressions without curing the internal condition *is a true suppression* and the *prolific parent of future ill health.*

As illustrative of these principles, a case of diphtheria may be cited; and what do we see therein? A person has been exposed to the contagion of diphtheria, and after four or five, or even ten days, the period of incubation, he complains of lassitude and dullness, followed by more or less chilliness and succeeding fever, backache, and general muscular pains and sore throat, which, on examination, exhibits the characteristic white or gray exudation, and the patient is said to have diphtheria; but he had diphtheria from the moment he received the contagion, a contagion so subtle that none of his senses could detect its approach, yet so virulent and progressive that the system became gradually surcharged, when, the vital forces concentrating their efforts for its *expulsion*, their appears on the tonsils or fauces "*an outward and visible*

*sign of this inward and invisible" evil.* What shall we do with this exudation? Shall we mistake it for the disease proper, and addressing our forces thereto, with probang, gargles, and caustic obliterate the excrescence? Alas for us and for our patient if we do! an outpost has been taken, but its extinction disables us from attacking the centre, the appearance, color, and location of such deposit being indispensable to a selection of *the* curative remedy, whose action for good will be indicated subsequently by the changes which it works on this appearance, until the whole disease within and without is annihilated. But we have removed the deposit, and vainly do we then interrogate nature for guidance; she will give no more answer to our questionings than a time-piece from whose face the hands have been removed will tell us the hour of day. Has, then, nothing been gained by our tactics? Verily, much, for should the patient's life be spared there will almost certainly follow, sooner or later, a standing memorial of our incompetence in one or more of a train of sequelæ, such as chronic pharingitis, difficult deglutition, enlarged tonsils, susceptibility to affections of the throat from the slightest change of temperature, degeneration of the kidneys, dropsy, even general paresis, all as supposed consequences of the diphtheria proper, but almost invariably due to our repressive treatment.

Given then a case of disease, we have an internal condition, as originating, and in due time an outward or sensational expression of it as secondary, which outward, objective, and sensational symptoms combined form the true and only guide for selecting our remedy; but being so chosen, will, under judicious administration, totally remove the entire malady, while tampering with mere external symptoms either to arrest or alter their character, or any other therapeutic medicinal procedure whatever, will disappoint our expectations by failing to secure health.

If the numerous patients who consult us for relief from chronic ailments be questioned as to the origin of their malady, we shall be told of their not having been well since they had a fever, pneumonia, erysipelas, diphtheria, scarlatina, cholera, or a fever and ague. My own answer to all such statements has ever been that the *diseases they refer to were never cured*, that *palliation or suppression had been mistaken for cure*, the primary symptoms having merely receded on the organism to evoke other forms of suffering not unfrequently beyond all our skill to cure.

In light of these principles, let us consider the immediate object of this paper—*Malarial Fevers as liable to suppression by abortive treatment.*

The transmission of malarial poison into the system is not usually apparent until the morbific principle has permeated the whole domain, and the well-known chill, fever, and perspiration make it manifest. Of its nature and origin we do not now inquire, a subject already dealt with by many, and especially by Dr. J. W. Dowling, of New York, whose able paper thereon he kindly sent me. It suffices that we admit its subtle and imperceptible invasion of the vital domain and the *non-recognition of its presence until a process for its expulsion is set up*, the chill representing a receding of the vital force preliminary to the reaction of fever, both culminating in the grand expulsive sweat, to be renewed at successive intervals until the enemy is dislodged and health returns.

We believe that no careful observer of nature will question our conclusion that the foregoing process is set up and sustained by the organism as *curative in its nature*, and all things being equal to the emergency, health must be the final result. I shall assume this position and make no deduction from its force to accommodate those who might reply, How very few possess the requisite vigor of body to wait for such a consummation. Their objection is admitted, but in no wise does it detract from what is assumed, that the process as set up is *essentially curative*, but if curative, *not to be disturbed or arrested by any means which fail to make such process unnecessary.* And here let me say emphatically that any medicinal agent capable of arresting a paroxysm which does not contain in its provings a totality of the characteristic symptoms of the case *can only induce a suppression*, in whose train will sooner or later follow either a renewal of the attack, demanding renewed administration of the repressive remedies, or a metastasis, the *legitimate and natural effect of all such measures.* In the former, frequent repetition of the medicine may, and almost certainly will, induce that shocking state of depraved health known as malaria cachexia, due, as I believe, *simply to repeated attempts with inapplicable or excessive medication to suppress the natural malarial manifestation of chill, fever, etc.*

While a metastasis will hardly fail to disorganize any organ to which the virus is determined—witness, as resulting from this cause, enlarged or endurated liver and spleen, persistent and agonizing neuralgia, ovarian and uterine engorgement, varicose veins, vertigoes, indeed, almost any form of suffering may be so induced—I am aware it will be maintained by some, who combat the disease with ponderous doses of inapplicable medicines bearing no relation to the totality of symptoms, that their success bespeaks for them a more favorable judgment; but I fear that, having arrested the paroxysm, these gentlemen rest satisfied with their procedure because no *immediate* indications of evil follow. Yet a seed which we place under ground will not put forth its germinal shoot the following day, and some having hard coverings lie dormant a long time ere the first sign of growth is apparent. So the malarial germs, whether bacteria or other minute organisms, when suppressed in their primary effort to induce a chill, etc., may lie long ere their power for mischief is manifested, and our failure to watch and wait on such cases leads to serious error of judgment. We are all familiar with the popular cry: "Doctor, I have had an attack of ague, and I want you to break the chill quick;" and the pressure of circumstances may prove a strong temptation to take the easy and well-trodden path with our patient. We may rest assured that, whatever difficulty lies in the true path, its end *alone* is health and honor; and not a few of the public are recognizing the truth of my assertion—all, indeed, who receive their instructions from the genuine disciples of Hahnemann, and their knowledge of such principles must often bring a blush on those who fail to put such principles into practice.

The following colloquy may bear thereon:

A neighbor afflicted with chronic bronchitis was passing along the outer wall of a cemetery when taken with one of his severe paroxysms of cough, and being exhausted with the effort, had leaned against the graveyard wall to recover breath. A sympathetic acquaintance from an opposite side having seen and heard his distress, crossed over to him with the ejaculatory advice:

"My dear sir, you should have that cough stopped."

"Stopped?" rejoined the sufferer. "Stopped, did you say? Believe me, sir, there are a good many lying on the other side of that wall who would give a heap for my cough."

"Ah," said his friend, slowly perceiving the point of his reply, "I see; that's a new view of the matter."

"Yes, sir, and a very important one; if a poor fellow has a lot of bad stuff in his chest he needs a pump to get it up. The cough's my pump, sir, which I cannot dispense with until my lungs are cured."

"Hem; good morning," and away went our sympathetic friend with the new idea to reflect on.

The chill from malarial infection bears the same relation to that poison which the cough does to our friend's loaded chest, and must on no account be arrested except by such means as *render its action unnecessary*—only possible under a potentized remedy, administered in accordance with the principles we have educed from the *Organon* of Samuel Hahnemann. By such an agent the morbific influence is neutralized at its *source*, and all the external manifestations, from the mild multifarious types of our northern regions to the pernicious chill of the south, yield to its god-like power as the darkness of night to the morning sun; moreover, the cure is complete and subsequent recurrence about as rare as second attacks of scarlatina or measles, and never owing to any imperfection in the original cure, but to a reproduction in the system of that morbific condition on and through which alone the malarial germs can again affect as injuriously. It is believed that the experience of every practiced homœopath will confirm the foregoing statements in illustration of which I add a few cases from my note-book which might have been multiplied had the limits of this paper allowed:

In the year 18— I was called on to visit a Mrs. D., age forty, who had came some forty miles to place herself under my care for the treatment of an obstinate and grave inflammation of both eyes, supposed to have arisen from cold, and which had hitherto resisted all attempts at cure. The inflammation was severe, and the eyes so extremely sensitive that an examination beyond a mere glance was out of the question, and I hesitated somewhat to assume the responsibility of their treatment.

Without delay she was placed under the use of such remedies as seemed indicated by the *ascertained* totality of the symptoms, the names of which, writing from memory, cannot now be recalled. This treatment continued for about three weeks, the only beneficial result obtained being a slight mitigation of the symptoms. Not satisfied with so poor a return, and diligently searching for some cause for this partial success, I conceived that the history of the case might not fully have reached me, so I sat down for a patient inquiry, from which was gathered that Mrs. D., with her husband, emigrated from the city of London some years before this, and had purchased a piece of land on our northern railroad contiguous to an extensive marsh, the proximity of which eventually induced recurring attacks of intermitting fever, for which Quinine had been freely and often taken, with the usual effect of at length "breading the chill," as it is termed, and, as our patient supposed, of curing the disease. Unfortunately, when the ague ceased its chill, etc., the eyes, which had hitherto been sound, became gradually inflamed, and so persistently and severe that at times total loss of vision seemed imminent. My inference from this statement was that the intermittent fever had not been cured by the Quinine, but suppressed and so thrown back into the system to concentrate its baneful effects in another form, which I conceived to be this affection of the eyes.

Should these deductions be correct, it was further premised that no improvement in the eyes was possible unless the restraining and suppressive action of Quinine on the primary disease should be antidoted, and if this were practicable the intermittent *might* return. Actuated by these thoughts and the presence of nausea as a prominent but *hitherto unrecognized symptom*, I gave Ipecac. 30 four times daily during several days, when, to my surprise and delight, one morning about nine a very decided chill set in, more severe than any which the patient had yet experienced, followed by intense fever and subsequent perspiration. Nat. mur. at once came into my mind as a probable remedy, but I waited for confirmation.

The next day was an intermission, succeeded on the third by a renewal of all the symptoms, time, etc., of the first. I had then a clear tertian beginning at 9 A. M., from which and other symptoms now forgotten there remained no reasonable grounds for neglecting Nat. mur. as *the* remedy. It was accordingly administered in the 30th potency four times daily for awhile, and after three paroxysms, occupying nine days, the disease ceased to return, being, as the sequel showed, completely cured; and to my great delight, the Natrum had acted so beneficially *nothing else was required*, and I shortly had the pleasure of sending home my patient cured of both the malarial fever and the terrible effects on the eyes of its having been suppressed.

Perhaps the most frequent result from such pernicious treatment is a liability on the part of the patient to some form of neuralgia. Many such consult me who have suffered thus during years. They usually yield to the carefully selected remedy for the totality, but the choice is often extremely difficult, arising from the imperfectly developed expressions, partaking, as they do, partly of the natural dynamic disease and partly of those symptoms which belong to the medicines which were used in its repression; and the genuine disciple of Hahnemann is not to be judged as swerving from our law of cure when in combating *poisonous doses of drugs* he administers at the outset a sensible dose of an antidote often on a mere chemical, principle, as the Sesquioxide of Iron or Arsenic, or the Albumen of eggs for Corrosive sublimate; but the chronic conditions induced can only be reached by the dynamic remedy and often require its highest forms. So far as my own experience goes, I have not found it necessary to deviate from the strict method of Hahnemann, yet there will occasionally be one who either fails of a cure or reaches it very slowly. I recall a sufferer of fourteen years from neuralgia, and burdened with almost every agent which varying physicians could give her, who remains to this day after some six months of my own treatment, though greatly relieved and only rarely in suffering, not cured, as shown by a recurrence of attacks from slight causes. Another, to whom Quinine has been given enormously for years, and who was consigned to her grave by three medical advisers, has so far yielded to the dynamic treatment that her chill and fever are restored, having now weekly paroxysms leaving an intermission of six days of fair health. Every well-chosen remedy has acted on her most satisfactorily, but a new type follows each success, demanding fresh consideration and a new remedy. These ravages in the human economy from the careless administration of Quinine, bring in mind the ominous warning of the master: "Wherever China is given for any condition of debility not induced by loss of fluids, and which is not the disease itself, its exhibition may be followed by the most pernicious consequences, and may even endanger life. Indeed, even in these cases it produces an excitement; but it is not a natural excitement; it is an overstraining of the vital powers of the patient, which may be followed by a perfect collapse, or may entail upon the patient a cachectic condition of the system which it is either difficult or frequently impossible to cure." What would this profound mind say could he witness the present wholesale abuse of this valuable drug? What, with our quinine tonics, quinine wine, quinine ale, and the endless preparations of quinine and iron, quinine and pepsin, we have fallen on evil times, and the pure doctrines of Homœopathy become emasculated by alliances with any conceivable pathy going. Every good gift and every perfect gift cometh down from above, from the Father of Lights, while all else is from beneath, from the Father of Lies, and as no lie is of the truth, so no truth is from the Liar. It may therefore be profitable that we inquire from whence our inspiration is drawn. If from beneath, whether knowingly or in ignorance, we are in danger of verifying in our experience the old adage that "What is got over the devil's back will be spent under his belly."

